# A Facile Measurement for Monitoring Dragline Silk Dope Concentration in *Nephila pilipes* upon Spinning

**DOI:** 10.3390/ma11101951

**Published:** 2018-10-12

**Authors:** Hsuan-Chen Wu, Shang-Ru Wu, Thomas Chung-Kuang Yang, Jen-Chang Yang

**Affiliations:** 1 Department of Biochemical Science and Technology, National Taiwan University, Taipei 106, Taiwan; hcwu7@ntu.edu.tw (H.-C.W.); r05b22057@ntu.edu.tw (S.-R.W.); 2 Research Center for Biomedical Devices and Prototyping Production, Taipei Medical University, Taipei 110, Taiwan; 3 Department of Chemical Engineering and Biotechnology, National Taipei University of Technology, Taipei 106, Taiwan; ckyang@mail.ntut.edu.tw; 4 Graduate Institute of Nanomedicine and Medical Engineering, College of Biomedical Engineering, Taipei Medical University, Taipei 110, Taiwan

**Keywords:** spider, silk, major ampullate gland, *Nephila pilipes*

## Abstract

In spite of all the efforts towards deciphering the silk spinning process of spiders, the underlying mechanism is yet to be fully revealed. In this research, we designed a novel approach that allowed us to quantitatively evaluate the concentration change of silk dope during the liquid-to-solid spinning process of the orb-weaver *Nephila pilipes*. As a prior characterization of the optimal silking conditions, we first gauged the influence of silking-rate, ranging from 1.5 to 8.0 m/min, on dragline silk diameters and silk tensile strengths obtained from the spiders. Next, to evaluate the liquid content of the silk dope, the major ampullate gland was dissected and the concentration of the sac portion was measured by thermogravimetric analysis (TGA). The solid content of the dragline fibers leaving the spinneret was investigated by calculating the ratio of collected dried silk to the weight loss of the spider recorded in situ upon spinning. As the results indicate, the tensile strength and diameter of the spun dragline fibers were 800–1100 MPa and 8–11 μm, respectively. The liquid content of silk stored in the major ampullate sac (50.0 wt%) was significantly lower than that of silk leaving the spinnerets (80.9–96.1 wt%), indicating that a liquid supplying mechanism might be involved during the spinning process. This reveals, for the first time, quantitative evidence in support of the lubricative hypothesis proposed formerly, namely that a liquid coating layer is supplemented to compensate for silking resistance during the spinning process of a spider. The spigot, at the exit of the spinneret, is speculated to serve as a valve-like controller that regulates the lubrication process along with fiber formation. Taken together, these findings provide understanding of the physiological functions in the spider spinning process and could further shed some light on the future biomimetic development of silk material fabrication.

## 1. Introduction

Studies of spider silk fibers are largely motivated by the exceptional strength, toughness, biocompatibilities, and potential medical applications of the fibers [1,2,3,4,5]. In spite of significant efforts to mimic native spider silk fibers, the mechanical performance of artificially-generated fibers, spun from either recombinant or natural silk, are often far below the expectation of super-strong fibers [2,4,5,6,7,8,9,10,11,12]. The source of silk proteins is not the only crucial factor; the processing of the silk materials has also been considered as another indispensable step towards creating high-performance silk fibers [11,13,14,15]. Spiders harness unique and eco-friendly processes, involving aqueous system and adequate temperature, for spinning their silk fibers [16,17]. Understanding the silk spinning process of spiders, the naturally born spinners, could provide an essential piece of the blueprint for the fabrication of novel artificially spun materials with superior properties. 

To date, many biomimetic spinning processes could hardly recreate the native spider silk spinning machinery inside the spider’s body, and the inferior physical properties of those resulting artificially-spun fibers are generally reported [18]. There remain efforts to bridge the technical gap, especially from the perspective of mimicking the physiological function of a spider’s duct as well as the liquid silk transition within it. The typical ampullate gland consists of a tubular tail portion, a sac-like midpiece, and a thin looped spinning duct linking the fluid silk reservoir with the spinneret. The mechanism of silk fiber formation, yet to be completely understood, is generally associated with shear stress induction, pH and ionic gradients, and silk dope concentration change inside the spinning duct [19,20]. It is also widely considered that the function of the spinning duct is to extract water out of the liquid silk of a spider [21,22,23,24,25,26] during its silking process. Particularly, Vollrath and Knight [6] examined the ultrastructure of a spinning duct and indicated that the duct’s wall behaves like a hollow fiber dialysis membrane that removes water from the liquid silk during spinning. The water-removal role of the spinning duct could indeed facilitate silk thread formation and liquid-to-solid silk transition. Subsequently, various mechanisms of liquid crystalline spinning [9], stress-induced phase separation [27,28], the model of shear-induced conformational change [29], and the element composition changes along the spinning duct during the formation of silk threads [19] were all soundly investigated.

The thread formation of a silk protein is usually governed by complex rheological viscoelasticity in the spinning duct. Prior to fiber spinning the fluid silk needs to be conveyed from the ampullate sac into a tiny but lengthy spinning duct [6]. The main issue is the flow resistance caused by the convergent flow of fluid silk around the funnel. The approximate pressure required to push liquid silk over the spinning duct can be estimated by the Hagen–Poiseuille equation [30].

(1) $$\Delta P=\mu L\times \frac{8Q}{\pi }\times R^{4}\;$$

where ΔP is the pressure drop, L is the length of the tube, R is half the diameter, and Q is the flow rate. Equation (1) shows the 4th-order influence of vessel radius on flow resistance for a Newtonian fluid within a tubular configuration. The diameter of the spinning duct shows greater than tenfold reduction relative to that of the sac-like mid-piece, such that the flow resistance in the duct could increase drastically, up to 10,000 times. Similar findings related to high flow resistance in spiders were also reported by Kojic et al. [31]. The sac does not, however, exhibit any observable muscle tissue to facilitate the expulsion of the secretions (personal observations) and compensate for the huge extrusion resistance. Additionally, water removal in the spinning duct that further increases the concentration and viscosity of silk dope could even intensify the obstacle of spinning. In summary, according to these arguments, there might exist alternative physiological pathways that dissipate the silking resistance built up along the spinning duct as well as promote the overall spinning efficiency. Therefore, a reevaluation of the spinning process of spider silk threads is highly desirable. 

In this study, we assessed the liquid/solid concentration of silk along the spinning system and at the silk entrance at the gland sac versus the silk exit at the spinneret as a clue to the overall liquid flow and silk physiology of the spider. This, in turn, will provide a beneficial means to elucidate the global spinning mechanism of spider silk proteins and will therefore also shed light on future artificial silking processes.

## 2. Materials and Methods

### 2.1. Spider Silk Collection and Measurement of Mechanical Properties 

Adult female *Nephila pilipes* (Fabricius 1793) (body length > 40 mm) were collected from low-altitude mountainous areas in Northern and Central Taiwan. In this study, we first examined the basic physical properties of their silks. Only dragline silks produced by major ampullate glands were utilized. To collect the dragline silk, we pulled the silk from the spinnerets of a secured spider under a dissecting microscope then taped the two threads on a rotor powered by a motor [32]. A digital winder controlled the take-up speed, ranging from 1.6–8.0 m/min. The fineness of silk fibers was first measured using Vibroscope-400 (Lenzing Instruments, Gampern, Austria) with the unit of denier, then converted into μm diameter by taking the silk density of 1.24 g/cm^3^ into consideration. The silk fiber tenacity was obtained with a tensile tester ZWICK 1445 (Zwick Roell, Ulm, Germany). A gauge length of 25.4 mm with a crosshead speed of 100 mm/min was utilized. For each tensile measurement, 20 silk filaments were used and all of the results were subsequently averaged to obtain representative data. The ambient humidity and temperature were maintained at ~40–60% and 26–28 °C, respectively, throughout the whole experimental period. 

### 2.2. Measurement of Liquid Content of Silk Fibers Exiting the Spinneret

Each *Nephila* *pilipes* spider was first secured on cardboard placed on an analytical balance (AG204 Delta Range, Mettler Toledo, Greifensee, Switzerland) with a readability of 0.01 mg. The balance was connected to a personal computer to collect the real-time weight change of the spider during the experiment. Prior to silking, the weight loss of the spider was recorded for 30–60 min at the resting condition, and the metabolic weight change was acquired. The major ampullate gland silk threads of the same spider were then harvested using the reeling rotor for 30–90 min, and the in situ and real-time weight change of the same spider during forced-silking was traced. Subsequently, the collected silk threads were further dehydrated in an oven and the dry weight was quantified. Finally, the liquid content of silk immediately after extrusion from the spinneret can be calculated by the following equation:

Liquid content (%) = [1 − W_dry silk_/(W_body weight loss_ − R_metabolic rate_ × T_silking_)] × 100 (2)

where W_dry silk_ designates the dry weight of silk drawn from the spider, W_body weight loss_ designates the body weight loss of a spider during the forced-silking process, R_metabolic rate_ designates the average weight loss rate of the spider due to metabolism, and T_silking_ designates the time period of silking. A total of five biological replicates were performed with separate spiders followed by the liquid content estimation. 

### 2.3. Measurement of Gland Silk Liquid Content 

The liquid content of the gland silk was estimated by a thermogravimetric analyzer (TGA-Q50, TA Instrument., New Castle, DE, USA). Major ampullate glands were dissected directly from female *N. pilipes* under a dissecting microscope, and the procured glands were gently wiped with Kimwipes to remove residual fluid from the outside of the glands. The shell of the silk gland was carefully opened and then removed with tweezers. The weight loss of fluid silk during heating was monitored, with the heating temperature ranging from 25–600 °C. During the initial stage of continuous heating, the evaporation of liquid resulted in a plateau of the mass loss value and thus the ratio of dehydrated silk to liquid was estimated.

## 3. Results

**Figure 1:**
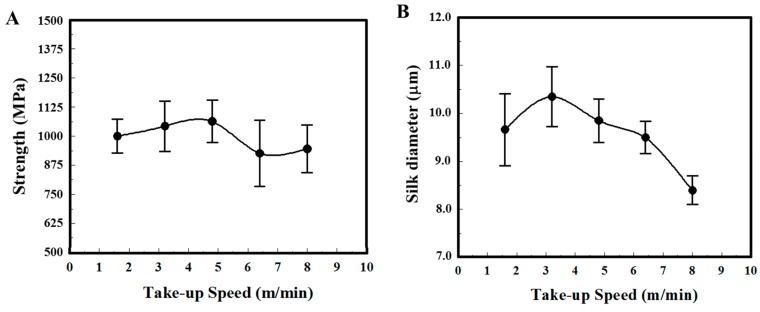
Physical properties of dragline silk from *N. pilipes.* (**A**) Strength (MPa) and (**B**) diameter (μm) under different take-up speeds.

Major ampullate dragline silks of *N. pilipes* were collected at varied winding speeds via a programmable reeling rotor. The physical properties of the silk samples, both fiber thickness and tensile strength, were systematically assessed (Figure 1). Figure 1A,B indicate the take-up speed dependence of silk fiber strength and diameter, respectively. The average silk fiber tenacity of *N. pilipes* was around 950–1060 MPa and the maximum value was between 1250–1500 MPa. No evident speed–tenacity relationship was observed in Figure 1A. On the contrary, the silk thickness drawn from the spiders seemed to be influenced by the winding speed (Figure 1B) [26]. At low-take up speed (<3 m/min), the diameter of silk threads slightly increased along with the spinning speed; the diameter of spun fiber gradually declined with spinning speed when the speed exceeded 3 m/min (e.g., 10 μm at 3 m/min versus 8 μm at 8 m/min).

**Figure 2:**
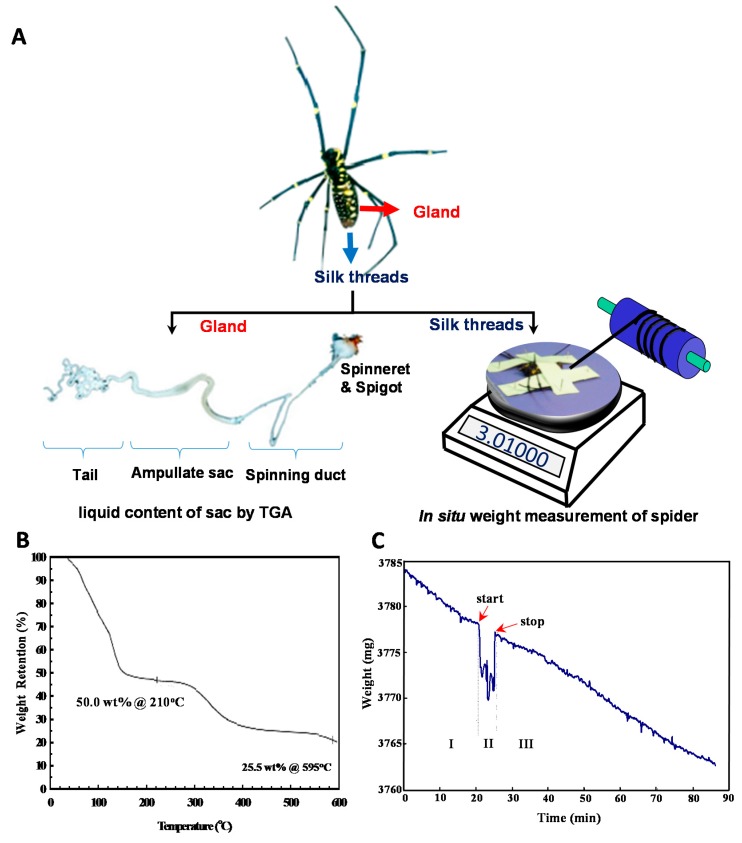
Measurement of spider silk concentrations along the spinning process of *N. pilipes*. (**A**) Schematic for assessing the liquid concentration of the silk dope at different locations in the spinning pathway. The concentration of silk in the gland sac harvested from a spider was estimated via thermogravimetric analysis (TGA) (**left**). The concentration of silk exiting the spigot was gauged by monitoring the weight loss of the spider on the microbalance and the weight of the collected dried silk (**right**). (**B**) The TGA weight percentage of the liquid silk sample shown in the left side of Figure 2A. (**C**) Real-time body mass change of the spider in the process of (I) pre-silking, (II) peri-silking, and (III) post-silking upon forced-spinning via the winder.

To gain more physiological insight into the silk spinning process of a spider, we measured the concentration change of silk dope along the spinning process. As shown in Figure 2, two approaches were utilized to gauge the concentration at both terminals of the silk gland system, namely the gland sac (the silk source) and silk fibers leaving the spinneret (the silk drain). Briefly, as shown in the left of Figure 2A, a major ampullate silk gland was isolated from the abdomen of a dissected *N. pilipes*. Similar to other *Nephila* spiders, the ampullate silk gland of *N. pilipes* also consists of a tail, a sac-like mid-piece, and a thin looped spinning duct, which links the sac to the funnel. We then further analyzed the solid/liquid content of the amupllate sac portion via thermogravimetric analysis (TGA), and the result is shown in Figure 2B. During heating, the sample mass loss reached the first plateau (50.0 wt%) when the temperature exceeded 100 °C. The level-off of mass during this stage of heating was most likely a result of liquid evaporation. When the heating temperature exceeded 300 °C, thermal degradation occurred. 

As depicted in the right of Figure 2A, a novel strategy was implemented for an accurate evaluation of the liquid content of silk exiting the spigot of the spider’s abdomen. The quantity of liquid carried by the silk fibers exiting the body was indexed by subtracting the amount of dried silk from the spider weight change associated with the silking process. Specifically, the spider was securely immobilized on the microbalance with the major ampullate silk fibers attached to the reeling winder. The body weight of the spider was then continuously monitored in situ. As the example in Figure 2C demonstrates, the change in the body weight of the spider was recorded from before the forced-silking process to after its completion. The real-time weight loss curve was divided into three states—state I, II, and III—which indicated the pre-, peri-, and post-silking processes, respectively. In states of I and III, the secured spider was kept statically on the microbalance without experiencing the dragging force exerted by the winder. The weight decreased monotonically at the average rate of ~0.3 mg/min, the respiratory metabolic rate of the spider [33,34]. In state II (the silking period), the dragline silk was drawn by the controlled winder and the reading of the microbalance decreased abruptly, owing to the disturbance of the external force. Nevertheless, the mass reduction of *N. pilipes* was obtained by estimating the weight loss that occurred during state II, from the start to the end of silking. The silk fibers collected from the winder (Figure 2A) were harvested and dried in an oven and the solid content of those desiccated fibers was gauged subsequently. Finally, the actual amount of liquid associated with the silk fibers was estimated by Equation (2), which represents a quantitative means for probing the percentage liquid content of silk fibers in situ.

Table 1 summarizes the detailed description of both the liquid and solid contents of silk fibers from *N. pilipes* spiders. Five replicate measurements were independently conducted with individual spiders. The estimated metabolic rates were ~0.3–0.5 mg/min and the liquid contents of dragline silk leaving the spinneret ranged from 80.9% to 96.1%. This, in turn, suggests an overall higher liquid content of silk dope at the spinneret than that of silk in the gland sac, ~50.0 wt%. 

## 4. Discussion

Over 46,856 spider species have been identified globally [35] and such a diverse spider population would offer a valuable resource for us to investigate the process of silk-associated spinning. In this study, we implemented a series of spider screenings and selected the golden orb-weaver *N. pilipes* as our model species because of its abundance in population, ease of handling, and possession of silk with strong mechanical properties [36]. *N. pilipes* is among the largest orb-web builders worldwide, especially in Taiwan, and has a body length is in the range of 30–50 mm. The maximum amount of silk obtained from a healthy adult can sometimes reach 5–15 mg in a single harvest using the forced-silking approach [37]. The average silk fiber tenacity of *N. pilipes* (950–1060 MPa) is comparable to that of *N. clavipes*’ dragline silk [9,36]. 

Controlling the dragline fiber thickness in a spider involves complex regulatory machinery within the spinneret. Our forced-reeling approach provided a means to further reveal the regulation. From Figure 1, we speculate such a thickness pattern may result from the internal regulation of the spinneret to release the pressure caused by forced-silking on the spiders. Vollrath and Knight [9] suggested that there is a valve system at the exit of the spinneret that is regulated by muscular action and could vary the thickness of the silk thread. Based on that assumption, we further hypothesize that only when the take–up speed exceeds a threshold beyond the spider’s internal regulation capability (e.g., >3 m/min), might the diameter of spun fiber be reduced with spinning speed. In nature, a spider could easily apply high spinning speed by administering sufficient external shear forces, dragging the silk through its own leg or descending via gravity, to produce the silk threads it needs [26]. 

The emphasis of this research was mainly to elucidate the physiological regulation of silk concentration along the spinning apparatus of a spider. From the perspective of material mass balance, we regarded the silk gland as an open system and probed the silk and liquid concentrations at both ends. The liquid content of silk dope inside the ampullate sac (designated as the system inlet) was estimated to be ~50.0% (Figure 2B), similar to that of *N. clavipes* [38]; on the other side, the liquid content of the silk threads exiting the spigot (designated as the system outlet) was calculated to be 80.9–96.1% (Table 1). As noted in Equation (2), the devised estimation, we believe, is able to approach the genuine liquid content of spider silk right at the exit of the spinneret upon spinning. The calculation is simply based on the material balance of liquid in respect to two interacting elements: silks and spiders. Namely, the total weight loss of the spider during silking equals the summation of silk loss, liquid loss and spider metabolism. Therefore, the liquid portion can be accurately assessed by considering those factors. 

As demonstrated in Table 1, our study focused on evaluating changes in the liquid content of silk immediately after it was drawn from the spinneret, and thus it avoided biased measurements due to uncontrolled liquid evaporation from the silk fibers over time. We propose that the majority of liquid co-spun with the silk threads would be quickly lost once the moisture is exposed to the ambient environment, due to the high surface-to-volume ratio of the dragline silk (microns of thickness). Liquid removal by evaporation only takes place after the silk exits the spinneret and can serve as an important mechanism for facilitating silk thread formation. 

**Figure 3:**
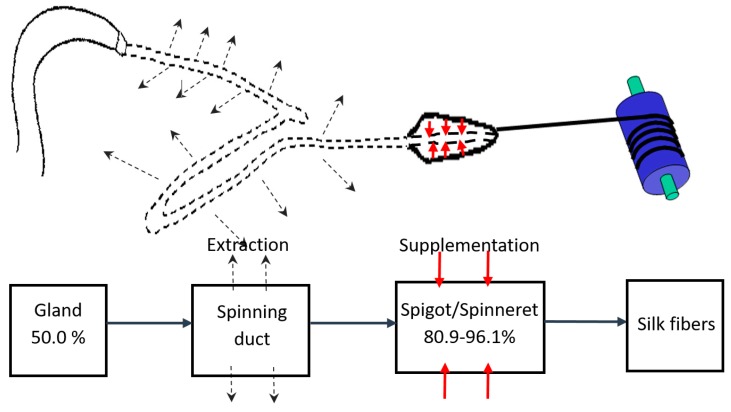
Schematic of the proposed major ampullate silk spinning model for *N. pilipes.* The liquid content of the silk dope initially decreases as it passes through the spinning duct (liquid extraction; dashed line), and then increases again at the spigot (liquid supplementation; red line) upon silk thread formation.

Furthermore, the increase in the liquid content of the silk dope along the spinning process, from ~50.0% at ampulla sac to 80.9–96.1% at the spigot, implies that a liquid supplementation mechanism might be involved in the spinning process. Complementary to the liquid extraction model for the spinning duct of a spider, we speculate there is a liquid addition mechanism at the distal portion of the spinning duct, adjacent to the spigot [9]. The extra-liquid supply mechanism, in turn, could abrogate the huge silking resistance of silk dope that results from the tapered spinning duct, as well as hardened silk threads upon silking. Furthermore, the added liquid layer applied to the spider silk threads could also serve as a protective coating to alleviate any potential damage to the lining layer of the spinning duct upon silking. As shown schematically in Figure 3, a highly facile spinning process is proposed in two steps: (1) liquid silk dope stored in the ampulla sac is first drawn through the spinning duct where the liquid-to-solid silk transition is expedited by water removal and ion exchange; and (2) liquid supplementation then occurs at the spigot to reduce the silking resistance and further facilitate silk thread extrusion.

To sum up, we offer evidence to elucidate the working hypothesis for the spigots of a spider, which can macroscopically supplement liquid into the silk dope as it is transported from the sac to the spinneret. The novel findings presented here might provide greater understanding of spider silk physiology as well as alternative insights to advance the current spinning technologies for man-made spider silks. 

## Figures and Tables

**Table 1:** Summary of liquid and solid content estimation on major ampullate dragline silk immediately drawn from the spigots of female *N. pilipes* spiders.

	Spider	No. 1	No. 2	No. 3	No. 4	No. 5
Item						
Weight of spider (mg)		3041.0	2850.6	3013.6	2698.1	2873.1
Weight loss during silking (mg)		12.0	15.5	44.8	33.1	12.6
Metabolic rate (mg/min)		0.3	0.3	0.4	0.5	0.3
Silking time (min)		29.0	25.0	31.0	23.0	30.0
Weight of dried silk (mg)		0.5	0.7	1.3	1.2	0.6
Silk conc. exiting spinneret (wt%)		19.1	7.4	3.9	5.4	12.0
Water conc. exiting spinneret (wt%)		80.9	92.6	96.1	94.6	88.0
